# Young people who are being bullied – do they want general practice support?

**DOI:** 10.1186/s12875-016-0517-9

**Published:** 2016-08-22

**Authors:** Emma Scott, Jeremy Dale, Rachel Russell, Dieter Wolke

**Affiliations:** 1 Warwick Medical School, University of Warwick, Coventry, CV4 7AL UK; 2 Department of Psychology, University of Warwick, Coventry, CV4 7AL UK

**Keywords:** General practice, Bullying, Children, Parents, Questionnaire, Internet

## Abstract

**Background:**

Childhood bullying is a major risk factor for health, education and social relationships, with effects persisting into adulthood. It affects half of all children at some point, with 10–14 % experiencing bullying that lasts for years. With the advent of cyberbullying, it can happen at all times and places. There have been calls for GPs to take a more active role in identifying and supporting young people who are being bullied. This paper explores young people’s and parents’ opinions about whether general practice should be involved in identifying and supporting young people who are being bullied.

**Methods:**

Two hundred six young people (85.9 % female, mean ± sd age 16.2 ± 3.2 years) and 44 parents were recruited through established bullying charity websites and their social media channels to complete an online questionnaire comprising multiple-choice questions and unlimited narrative responses. Questionnaire responses were analysed by age and gender using descriptive statistics. A descriptive analysis of the narrative responses was undertaken and key themes identified.

**Results:**

Young people (90.8 %) and parents (88.7 %) thought it was important for GPs to be better able to recognise and help young people who are being bullied. Most recognised the link between bullying and health. The doctor’s independence was seen as advantageous. Young people preferred completing a screening questionnaire to disclose experience of being bullied than being asked directly. They expressed concerns about how questions would be asked and whether information would be shared with parents/guardians. Parents were supportive of the use of a screening questionnaire, and most expected their child’s disclosure to be shared with them.

**Conclusion:**

Young people and parents recruited through anti-bullying websites and social media would welcome greater GP involvement in identifying and supporting young people who are being bullied and their families, provided it is offered in a caring, compassionate and confidential manner.

## Background

Bullying is a systematic abuse of power characterised by repeated psychological or physical aggression with the intention to cause distress to another person. Over half of young people (55 %) report having recently been bullied [1], with 10–14 % experiencing chronic bullying lasting for more than six months [2]. Bullying occurs at similar rates across all socio-economic strata [1, 3] with both minority ethnic and white youths reporting comparable levels of victimisation [4]. Although often perceived as a school-based problem, bullying is increasingly community-based. Social networking sites and smart-phones have brought with them a new phenomenon – cyber-bullying, which can happen at all times and in all places [5]. Recent figures show that 15 % of 15 year olds in the UK have experienced cyber-bullying. Girls are more likely to experience psychological, emotional and cyber-bullying, whereas boys are more likely to be physically bullied [1].

Childhood bullying is a major risk factor for health, educational attainment and social relationships. Bullied children are twice as likely as non-victims to suffer from psychosomatic problems, such as headaches, abdominal pain, sleeping problems, poor appetite and enuresis [6]. They are at increased risk of psychiatric disorders including depression, eating disorders, self-harm and suicidal behaviour [7, 8]. They also have high rates of poor academic performance resulting from absenteeism and worries at school [9, 10]. Over 16,000 young people in the UK aged 11–15 years are estimated to be absent from state school with bullying as the main reason, and a further 78,000 are absent where bullying is one of the reasons given [11]. The adverse consequences of childhood bullying continue into adulthood leading to substantial health and wider societal costs. This includes difficulties with employment and social relationships, and mental health consequences such as general anxiety disorder, panic disorder, agoraphobia, depression, and suicidal acts [12].

General Practitioners (GPs) in the UK can offer supportive counselling either within the practice or from a third sector agency specialising supporting young people or bullying. They also have access to a range of other resources as recommended by the Royal College of General Practitioners (RCGP) [13]. If bullying is affecting the young person’s education, the GP can refer them to the educational psychology service or to Children and Adolescent Mental Health Service (CAMHS) if there is evidence of severe mental health issues. In addition to this, GPs are optimally placed to identify and treat the physical and psychological consequences of bullying outlined above. Talking to someone about bullying is the first step to getting help, but up to 40 % of children never disclose bullying to their parents [14]. Hence, the opportunity to discuss bullying with a healthcare professional may provide an important avenue to break the silence and initiate help.

Given the impact on health, children who are being bullied are likely to have greater need for health care than their non-bullied peers. Although research to confirm the extent to which this leads to more frequent attendance at general practice is lacking [15], previous work with school nurses has confirmed that there is a positive correlation between self-reported health symptoms (e.g. poor sleep, frequent headache) and frequency of bullying experienced [16, 17]. In the UK, school nurses do not typically consult with every student in the school every year. In countries, however, such as Denmark, where an annual consultation is routine, students who are being bullied are more likely to report positive effects of their dialogue with the school nurse and to initiate additional visits to the nurse [18]. The WHO has called for society-wide inter-agency approaches that include primary care and mental health services [19]. This has been echoed by NICE who include the evaluation of bullying as a risk factor and the development of anti-bullying strategies in several of its guidance documents, including depression [20] and weight management in young people [21] which specifically mention general practice. The Anti-Bullying Alliance, in collaboration with the Royal College of General Practitioners, marked Anti-Bullying Week 2015 with the publication of guidance notes for GPs [22].

Despite the calls for greater health service involvement, the extent to which young people and their families see bullying as a health issue relevant to general practice is unknown. This paper reports on work exploring the views of young people and parents about GPs taking a more active role in identifying and supporting young people who are being bullied.

## Methods

### Recruitment

Four national UK-based bullying charities (*Anti-Bullying Alliance, BeatBullying, Bullies Out, Kidscape*), which offer support to young people aged 11-25years, posted brief information on their websites, Facebook pages and twitter feeds, inviting young people who had experienced bullying to complete a brief survey. A hyperlink was provided to a page on the University’s website where more detailed information about the study was given, together with a link to a confidential survey page.

There was no direct contact between researcher and participant. Parental consent was not obtained for the young people participating. Clicking the link given in the invitation from the charity and then completing the online survey after reading the age appropriate information supplied about how their responses would be used, was considered to be implied informed consent. The reason for not seeking parental consent was to ensure that potential participants who have not disclosed the fact that they are being bullied to a parent were not unfairly excluded from the survey. There are many reasons that young people may not tell their parents that they are being bullied and it is particularly important that their opinions on other sources of support are heard. Our institutional ethics review board approved this approach and the rationale behind it.

A parallel request was made for parents of children who had been bullied to complete a similar survey. No active effort was made to recruit parent-child dyads. Parental participation was not contingent on their child also completing the questionnaire and no information was collected to match parent and child participants. Completion of the survey, after reading the introductory information about how their responses would be used, was considered implied informed consent.

The study received ethical approval from the University’s BioSciences Research Ethics Committee.

### Data collection

For young people, the survey comprised three multiple choice questions with free text space under each question. The questions were intended to stimulate interest, with topics covering the perceived importance of GP involvement in identifying and supporting children who are being bullied, and whether they would be comfortable with (a) their GP asking about bullying and (b) completing a screening questionnaire which included questions on bullying, in the waiting room prior to seeing the GP. The questions and answer options are presented in full in Table 1.

**Table 1:** Young people’s responses to the multiple choice questions

Q1: How important do you think it is for GPs to be better able to recognise and help young people who are affected by bullying?				
Very important	Quite important	Not Sure	Not very important	Not important at all
* n* = 109, 52.9 %	*n* = 78, 37.9 %	*n* = 11, 5.3 %	*n* = 7, 3.4 %	*n* = 1, 0.5 %
Q2: As a young person, how would you feel if a GP asked you about experiences of being bullied if you were attending the GP for an everyday problem such as a headache or tummy ache? Would you feel comfortable with this?				
Yes, completely	Yes, a bit	Not sure	Not very much	Not at all
* n* = 36, 17.5 %	*n* = 72, 35.0 %	*n* = 50, 24.2 %	*n* = 36, 17.5 %	*n* = 12, 5.8 %
Q3: We are thinking of asking young people to complete a questionnaire while in the waiting room when they visit the doctor to ask about their current health. This would include some questions about their experience of being bullied. Would you feel comfortable answering such a questionnaire in the waiting room?				
Yes, completely	Yes, a bit	Not sure	Not very much	Not at all
* n* = 100, 48.5 %	*n* = 68, 33.0 %	*n* = 15, 7.3 %	*n* = 17, 8.3 %	*n* = 6, 2.9 %

The parental survey had eight questions, three of which related to the bullying experienced by their child. The remaining questions covered the perceived importance of greater GP involvement, feelings about their child being asked to complete a screening tool for bullying, whether they thought their child would be honest about bullying if the GP asked and their experience of discussing bullying with their own GP. The questions and answer options are presented in full in Table 2.

**Table 2:** Parents’ survey responses

Q1: Has your child ever been bullied?						
Yes		No			Unsure	
* n* = 38, 86.4 %		*n* = 4, 9.1 %			*n* = 2, 4.5 %	
Q2: If you answered yes to Q1, what type of bullying was it? You may choose more than one option ^a^						
School	Outside school	Cyber	Emotional	Physical	Psychological	Other
* n* = 38, 86.4 %	*n* = 18, 40.9 %	*n* = 11, 25.0 %	*n* = 20, 45.5 %	*n* = 14, 31.8 %	*n* = 13, 29.5 %	*n* = 2, 4.5 %
Q3: Was your child aged 16 years or younger?						
Yes – primary school		Yes - secondary school	Yes – both schools		Yes – age not given	No
* n* = 10, 22.7 %		*n* = 10, 22.7 %	*n* = 4, 9.1 %		*n* = 20, 45.5 %	*n* = 2, 9.1 %
Q4: Do you think it is important that GPs should be better able to recognise and help young people being affected by bullying?						
Very important		Quite important	Not Sure		Not very important	Not important at all
* n* = 31, 70.5 %		*n* = 8, 18.2 %	*n* = 5, 11.3 %		*n* = 0	*n* = 0
Q5: How would you feel if your child was asked to complete a questionnaire while in the doctor’s waiting room which covered questions about their current health including their experience of being bullied?						
Positive – would expect child to share answers		Positive – would not expect child to share answers	Not Sure		Negative – I don’t think this is appropriate	
* n* = 24, 54.5 %		*n* = 12, 27.3 %	*n* = 3, 6.8 %		*n* = 5, 11.4 %	
Q6: If your child was being bullied do you think they would report this during a visit with a doctor if asked?						
Yes, definitely		Yes, maybe	Not sure		No, probably not	No, definitely not
* n* = 6, 13.6 %		*n* = 18, 40.9 %	*n* = 9, 20.5 %		*n* = 9, 20.5 %	*n* = 2, 4.5 %
Q7: What kind of problem do you see bullying as? You may choose more than one option ^a^						
School		Health	Neither		Other	
* n* = 33, 75.0 %		*n* = 23, 52.3 %	*n* = 1, 2.3 %		*n* = 17, 38.6 %	
Q8: Have you ever discussed with a GP any incidents of bullying of your child and its consequences?						
Yes – GP helpful		Yes – GP not helpful	No		Not yet, but am considering	
* n* = 7, 15.9 %		*n* = 9, 20.5 %	*n* = 26, 59.1 %		*n* = 2, 4.5 %	

^a^Note: parents were able to select more than one type of bullying so percentages will not add up to 100 %

Age and gender were collected for participants in both surveys.

The questions in both surveys were initially developed in consultation with representatives from the *Anti-Bullying Alliance* and *Kidscape*. The response options were refined with input from local parents and young people with experience of bullying. There was no formal pilot test phase.

### Data analysis

For the multiple choice questions in both surveys, response frequencies were tallied. Chi-square tests were conducted to explore any differences in response by age or gender, with a *p-*value of less than 0.05 considered to be significant. All data analysis was conducted using IBM SPSS Statistics 22.

Free text responses were downloaded verbatim from the online survey output and initially collated by which survey question elicited the comment. The lead author familiarised herself with the responses before undertaking a descriptive analysis of the data. As this was exploratory work, an inductive approach was taken. The data was subjected to a process of complete coding and candidate themes identified. These themes were then refined, redundant themes removed and further themes added as identified during subsequent readings of the data. The relationship between the themes and participants’ responses to the multiple choice questions was considered during analysis. As some themes were common to more than one question, the data was collapsed and the findings presented by theme.

## Results

### Participant characteristics

**Fig. 1:**
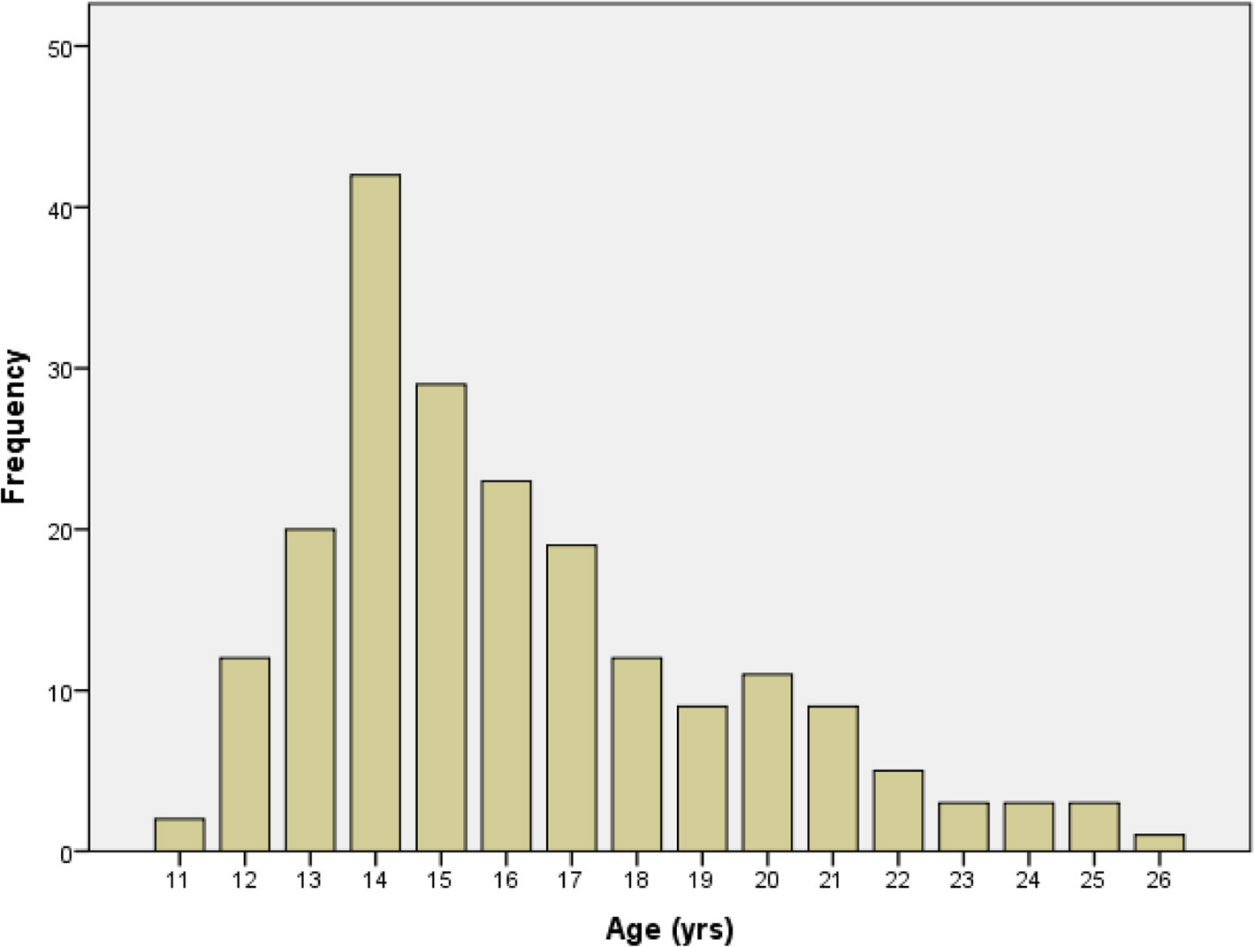
Age distribution of young people completing the questionnaire

Two hundred and six young people (85.9 % female, mean ± sd age 16.2 ± 3.2 years) and 44 parents (88.6 % female, aged 25–58 years) participated. The majority (55.4 %) of young people were aged 13–16 years (see Fig. 1), and there was no difference in gender distribution by age (*p =* .142).

Most parent participants (86.4 %) were certain that their child had been bullied, the others were less sure. Parents reported a wide range of types of bullying experienced by their child (see Table 2) with school bullying (86.4 %) being the most common. In the majority of cases (90.9 %) the child was aged 16 years or younger at the time.

### Quantitative data

All multiple choice questions received a 100 % response (see Tables 1 and 2). For young people, there were no differences in responses to any question by age (all *p* > .495) or sex (all *p* > .119).

#### Involvement of GPs

Most young people thought it important (very important 52.9 %; quite important 37.9 %) for GPs to be better able to recognise and help young people who are affected by bullying. Likewise, most parents also believed this to be important (very important 70.5 %; quite important 18.2 %).

Just over half of the young people described feeling comfortable (completely comfortable 17.5 %; quite comfortable 35.0 %) with their GP asking about bullying if they attended the surgery for a problem such as a headache. A quarter, however, would not be comfortable being asked (not very comfortable 17.5 %; not at all comfortable 5.8 %) and the remaining 24.2 % were uncertain. This pattern of responses was similar across all age ranges (*p =* .495).

Most parents recognised bullying as a school and/or health problem, with a significant proportion (38.6 %) identifying additional or alternative routes of the problem, i.e. social/community problem (*n* = 8), cyber-bullying (*n* = 2), family problem (*n* = 2), a self-esteem problem (*n* = 1), “a fact of life” (*n* = 1).

Over a third of parents (36.4 %) had discussed the bullying of their child and its consequences with their GP, but less than half had found it helpful.

#### Screening for bullying

When asked about completing a health questionnaire, which included questions about bullying, before seeing a GP, the vast majority of young people would feel comfortable doing this (completely comfortable 48.5 %; quite comfortable 33.0 %). A few were unsure (7.3 %) and some were not comfortable with this idea (not very comfortable 8.3 %; not at all comfortable 2.9 %).

The vast majority (81.8 %) of parents were positive about the idea of their child being asked to complete a screening questionnaire that included questions on bullying. Most would expect their child to show them their answers.

### Qualitative data

In total, young people provided 232 free text comments with a further 56 from parents. Most were quite brief (10–20 words) but others were notably longer (up to 700 words). Analysis identified four themes: Awareness of the link between bullying and health; The appropriateness of GP involvement; Confidentiality and the presence of parents; and, Practical issues surrounding screening.

#### Awareness of the link between bullying and health

Most young people and parents demonstrated understanding that bullying can be a cause or contributory factor in both physical and mental ill-health throughout the comments they provided. In many cases they spoke from experience and included personal examples. A minority, however, appeared unaware of bullying as a potential risk factor for common health concerns:

*“I don’t really see the link between GP and bullying. If I go in with a tummy ache or headache I would just want to get in there get medication and then come out.”* (Young Person #85, female, aged 14)

Experience of approaching the GP for support with bullying-related health problems was variable. GPs were perceived to lack understanding of bullying and its links to physical and mental health. Parents were particularly critical:

*“A medical problem may well be the first sign of bullying … it would be helpful if GPs were more aware of how prevalent bullying is and included it in any assessment of the child.”* (Parent #43, female, aged 52)

#### The appropriateness of GP involvement

Whilst both young people and parent participants were overwhelmingly in favour of GPs being better able to identify and support young people who are being bullied, they also expressed a number of reservations. A very small minority felt that tackling bullying was outside the doctor’s remit and should remain the responsibility of teachers and parents.

Both young people and parents thought GPs being removed from the school setting was an advantage. The doctors’ independence from both the family and school was considered beneficial and likely to allow a more objective assessment of the child and situation. In addition to this, young people felt it would be easier to talk to a more independent adult:

*“…it may be easier to talk to someone that they know probably doesn’t know the people they are talking about and that they won’t tell them.”* (Young Person #20, female, aged 14)

Over half of the parents surveyed thought that if their child was being bullied that they would probably tell the doctor, if asked, and those leaving comments identified the doctor’s approach to questioning as being key to facilitating disclosure. Parents and young people agreed that they would be more likely to report bullying if they understood why the doctor was asking (i.e. the link between bullying and health). Other key factors were GP sensitivity and offering reassurance:

“*As long as they were friendly and genuine I would quite happily talk about problems if someone was there to listen. I wouldn’t talk if it was spoken about in a generic way like a check mark against their daily tasks.”* (YP #176, female, 22)

Young people felt the most significant barrier to disclosure was the feeling that they didn’t have an established relationship with their GP. They expressed concern about their lack of connection with their doctor and the difficulties this may present in feeling safe talking to them:

*“You might not even want to tell an adult you trust, let alone one that you don’t really know”* (YP #138, female, 13)

Other concerns expressed by both young people and parents included whether GPs have the appropriate training and experience to deal with bullying and the time pressure of brief appointment slots.

#### Confidentiality and the presence of parents

A significant number of young people expressed a preference for the questionnaire being anonymous, but in the context of the comments it appears that there may have been some confusion between the terms ‘anonymous’ and ‘confidential’:

*“I know that this would be kept completely anonymous between myself and the doctor…”* (YP #20, female, 14)

While participants acknowledged the link between bullying and health and understood that the doctor would be trying to help, some felt that being asked about bullying might be uncomfortable or awkward, but could offer a means of relief:

*“I would personally feel weird and in an awkward position. However if one person does know about my situation they may help me and I may not be a victim any more.* (YP #9, female, 14)

Discomfort was expected to be greater if their parents were present and a few young people questioned whether children would disclose bullying in their parent’s presence. Many young people expressed a preference for parents/carers not to be present during discussions about bullying.

Most parents, however, expected their child’s disclosure to be shared with them and some expressed a desire to be the first person to help their child. Others gave a more balanced view about providing support to the child:

*“Would hope that my child would share info with me, but it is important that they know it would be confidential if they wish.”* (Parent #43, female, 52)

#### Practical issues surrounding screening

Participants identified a number of practical issues regarding the use of a screening questionnaire to identify young people who are being bullied including delivery format and venue. There was a strong preference among young people for initially answering questions about bullying in a paper or online questionnaire rather than verbally face-to-face:

*“I think this would be a more suitable and effective way of approaching this topic … it’s easier to write things down than speak to someone”* (YP #92, female, 17)

Young people’s opinions were divided on the appropriateness of completing the questionnaire in the GP’s waiting room. Some participants felt that this was a good way of asking and that completing the questionnaire in the waiting room would result in better engagement.

*“I would feel comfortable answering a questionnaire in the waiting room as it gives me something to do while I’m waiting … I think it would then be easier to speak to the GP about when you went in”* (YP #126, female, 16)

But others, while positive about the idea of completing the questionnaire, raised concerns about privacy in the waiting room and suggested alternatives, such as completing it at home or online.

Parental concerns were focussed on the possibility of the questionnaire causing distress to a child, while others questioned whether a child would complete the questionnaire honestly. It was observed that the questionnaire would only be of use if the doctor valued the questions and the responses provided.

## Discussion

Both young people and parents recognised the link between bullying and health, and would welcome greater GP involvement in recognising and supporting young people who are being bullied, providing this was done in a caring and compassionate way. Young people viewed the completion of a paper or online screening questionnaire prior to the appointment as preferable to initially being asked about bullying face-to-face; parents also found this approach acceptable. Parents and young people disagreed about whether parents should be present during the discussion about bullying.

These are important findings considering that up to 40 % of children never disclose bullying to their parents [14], but that talking to someone about bullying is the first step towards help. Thus confidential disclosure to GPs may provide an important avenue to break the silence and initiate help. This may be in the form of counselling or support from the GP to manage the physical and psychological consequences of bullying, or referral to specialist services such as the educational psychology service or CAMHS [13].

Given the preference expressed by young people to complete a screening questionnaire rather than being asked directly about bullying, alternative routes to follow up with young people identified through screening should also be explored. Previous research has found school nurses to be a viable way of identifying and supporting young people who are being bullied [16, 18]. For this model to work in the UK, school nurses would need to be a more regular fixture in schools so that they are a trusted face rather than an infrequent visitor. The participants in this study placed significant value on the doctor’s independence from the school. Hence, the GP practice nurse might also provide an appropriate source of support.

Both parents and young people expressed concerns about how the GP would facilitate disclosure of bullying. The qualitative findings suggest that GPs may need to be more attuned to the importance of (a) considering a young person’s experience of bullying as a risk factor for poor physical and mental health, (b) building a trusting relationship with their young patients, (c) ensuring that enquiries about bullying are made in a caring and compassionate way; and (d) that young people are given their full attention when talking about bullying experiences. While many GPs may feel that they already seek to do these things, it still remains that these are the areas where young people and parents see room for improvement.

The discrepancy between young people’s and parents’ views related to parental presence when questions about bullying were being asked needs to be addressed. Most parents expected their child’s responses to be shared with them, but many young people expressed a preference for parents/carers not to be present during discussions about bullying. Similar research conducted with GPs has shown that the presence of a parent may also affect their willingness to initiate discussion of a potentially sensitive topic [23].

### Strengths and limitations

To our knowledge, this is the first report of young people’s and parents’ perspectives on the involvement of GPs in identifying and supporting young people who are being bullied.

The use of brief questions accompanied by unlimited text boxes allowed participants to expand on their questionnaire answers and put them into context. This combined approach resulted in a richer and more illuminating data set than is usually possible using survey methods alone and reached a greater number of participants than would typically be possible for traditional qualitative work. The quantity of free text responses received was unanticipated and what was originally expected to be a brief quantitative survey became a mixed methods study.

Data analysis was carried out by a single researcher. While her perspective may have influenced interpretation of the data, the accompanying quantitative data from each participant clarifies their point of view (positive, negative, uncertain) on each question, thereby reducing the risk of misinterpretation.

Recruiting participants through established bullying charity social media channels was an effective means of involving individuals with a diverse range of experience. Distributing paper questionnaires through schools or youth groups would likely have drawn a minority of participants with first-hand experience of chronic bullying. The use of an online survey also allowed greater preservation of participant anonymity. The use of different online research methods in health services research has been discussed in detail elsewhere [24, 25].

The main limitation of the sample is that the extent to which it is representative of the wider population of bullied young people and their parents is unknown. As recruitment was undertaken through bullying charity websites, the sample may have been biased towards individuals who are already actively seeking support to cope with bullying and may therefore be more receptive towards alternative sources of support, such as GPs. Furthermore the sample was predominantly female and, although girls are more likely to be bullied than boys [1], the difference is not as pronounced as the imbalance in our sample. In addition to this, girls and boys typically experience different types of bullying [1] and we did not collect any information on the type of bullying experienced by the participants. Although we observed no differences in the responses recorded by age or gender on this occasion, this does not mean that the results are necessarily generalizable to all bullying victims. Further work is needed to explore the opinions of young males who have been bullied and efforts should be made to obtain data on the types of bullying experienced by participants.

## Conclusion

This study reinforces calls for greater GP involvement in preventing the long term health consequences associated with childhood bullying [15, 19, 26, 27], and provides evidence that young people and their parents would welcome this. It provides new knowledge that young people would prefer to complete a screening questionnaire in the waiting room before the consultation, rather than be asked about bullying face-to-face. It is important that any future screening or support programme involving general practice would need to be acceptable to the intended recipients with confidential screening being a potentially acceptable way.

The focus of this paper is whether young people who are being bullied and their parents want GP support. Further work is also needed to explore how that support should be provided.

## Abbreviations

CAMHS: Child and Adolescent Mental Health Services
GP: General Practitioner
NICE: National Institute of Health and Care Excellence
RCGP: Royal College of General Practitioners
UK: United Kingdom
WHO: World Health Organisation
YP: Young Person
